# Supplementary material for: Metabolic imaging across scales reveals distinct prostate cancer phenotypes
Source: Nat Commun. 2024 Jul 16;15:5980. doi: 10.1038/s41467-024-50362-5 (PMC11252279; doi:10.1038/s41467-024-50362-5)
Supplement: Supplementary file 1 — Supplementary Information [file 41467_2024_50362_MOESM1_ESM.pdf]

Metabolic imaging across scales reveals distinct prostate cancer phenotypes

Nikita Sushentsev[1†*], Gregory Hamm[2†], Lucy Flint[2], Daniel Birtles[2], Aleksandr Zakirov[3], Jack Richings[2], Stephanie Ling[2], Jennifer Y. Tan[2], Mary A. McLean[1,4], Vinay Ayyappan[1], Ines Horvat Menih[1], Cara Brodie[4], Jodi L. Miller[4], Ian G. Mills[5,6,7,8], Vincent J. Gnanapragasam[9, 10,11], Anne Y. Warren[12], Simon T. Barry[13], Richard J.A. Goodwin[2††], Tristan Barrett[1††], Ferdia A. Gallagher[1††]

[1] Department of Radiology, Addenbrooke's Hospital and University of Cambridge, Cambridge, UK
[2] Imaging and Data Analytics, Clinical Pharmacology & Safety Sciences, R&D, AstraZeneca, Cambridge, UK
[3] Department of Clinical Neurosciences, University of Cambridge, Cambridge, UK
[4] Cancer Research UK Cambridge Institute, University of Cambridge, Cambridge, UK
[5] Patrick G Johnston Centre for Cancer Research, Queen's University Belfast, Belfast, UK
[6] Nuffield Department of Surgical Sciences, University of Oxford, John Radcliffe Hospital, Oxford, UK
[7] Centre for Cancer Biomarkers, University of Bergen, Bergen, Norway
[8] Department of Clinical Science, University of Bergen, Bergen, Norway
[9] Department of Urology, Cambridge University Hospitals NHS Foundation Trust, Cambridge, UK
[10] Division of Urology, Department of Surgery, University of Cambridge, Cambridge, UK
[11] Cambridge Urology Translational Research and Clinical Trials Office, Cambridge Biomedical Campus, Addenbrooke's Hospital, Cambridge, UK
[12] Department of Pathology, Cambridge University Hospitals NHS Foundation Trust, Cambridge, UK
[13] Bioscience, Discovery, Oncology R&D, AstraZeneca, Cambridge, UK

[†] These authors contributed equally
[††] These authors jointly supervised the work
*Corresponding author. Email: ns784@medschl.cam.ac.uk

Supplementary Information file including:

Supplementary Tables 1-2

Supplementary Figures 1-13

Peak	Interval width da	Metabolite
**87.0088**	0.00051129959854279	Pyruvate
**89.0246**	0.00052314529990838	Lactate
**103.0036**	0.00060529199649295	Malonate
**112.9877**	0.00066396247220268	Oxaloacetate
**115.0035**	0.0006758082859335	Fumarate
**117.0194**	0.00068765446259533	Succinate
**125.0972**	0.00073512268198073	Octanoic acid
**131.0351**	0.00077001646214114	Glutarate
**133.0145**	0.00078164818512505	Malate
**145.0144**	0.00085216473715377	alpha-Ketoglutarate
**152.9962**	0.00089906909218485	Succinate
**153.1287**	0.0008998474800137	Decanoic acid
**166.9755**	0.00098121705471499	Phosphoenolpyruvate
**173.0093**	0.0010166739553483	cis-Aconitate;Citrate
**179.0561**	0.001052207884598	Glucose
**189.004**	0.0011106654522308	Oxalosuccinate
**191.02**	0.001122512497858	Citrate
**208.9846**	0.0025771426214618	cis-Aconitate
**215.0325**	0.0012636201084888	Glucose
**224.9801**	0.001322076059239	Oxalosuccinate
**227.2014**	0.0013351295196173	Tetradecanoic acid
**241.012**	0.0014162866034297	Glucose 1-phosphate
**246.9398**	0.0014511207514829	Glycerate 1,3-biphosphate
**253.2173**	0.0014880098153043	Palmitoleic acid
**281.2482**	0.0016527310219772	Oleic acid
**283.2654**	0.0016645846906727	Octadecanoic acid
**284.056**	0.0016692306908794	S-Acetyldihydrolipoamide-E
**291.2099**	0.0017112699781592	Hexadecanoic acid
**317.2244**	0.0018641420160179	Oleic acid
**319.2406**	0.0018759897287168	Octadecanoic acid
**398.3282**	0.0023407415183669	L-Palmitoylcarnitine

Supplementary Table 1. The list of KEGG glycolysis, TCA cycle, and fatty acid biosynthesis metabolites used for DESI-MSI-derived metabolic pathway enrichment analysis in the secondary cohort.

Metabolite	Pathway	Ion Cluster	Calculated m/z	Measured m/z DESI	Error ppm	Measured m/z MALDI	Error ppm
Lactate	TCA cycle	$[M-H]^-$	89.024419	89.0243	0.3	89.0245	0.9
Arachidonic Acid	Fatty acid biosyn.	$[M-H]^-$	303.232954	303.2337	2.7	303.2322	2.5
Malate	TCA cycle	$[M-H]^-$	133.01425	133.0140	1.8	133.0142	0.4
Citrate	TCA cycle	$[M-H]^-$	191.01973	191.0205	4.0	191.0194	1.7
Palmitoleic acid (FA 16:1)	Fatty acid biosyn.	$[M-H]^-$	253.21621	253.2168	2.3	253.2164	0.8
Oleic acid (FA 18:1)	Fatty acid biosyn.	$[M-H]^-$	281.247507	281.2489	4.8	281.2486	3.9
Tetradecanoic acid	Fatty acid biosyn.	$[M-H]^-$	227.2014	227.2020	2.5	227.2009	2.2
Octadecanoic acid	Fatty acid biosyn.	$[M-H]^-$	283.26539	283.2633	7.3	283.2639	5.3

Supplementary Table 2. The list of comparative DESI-MSI and MALDI-MSI mass accuracy measurements for selected metabolites of interest.

Supplementary Figures

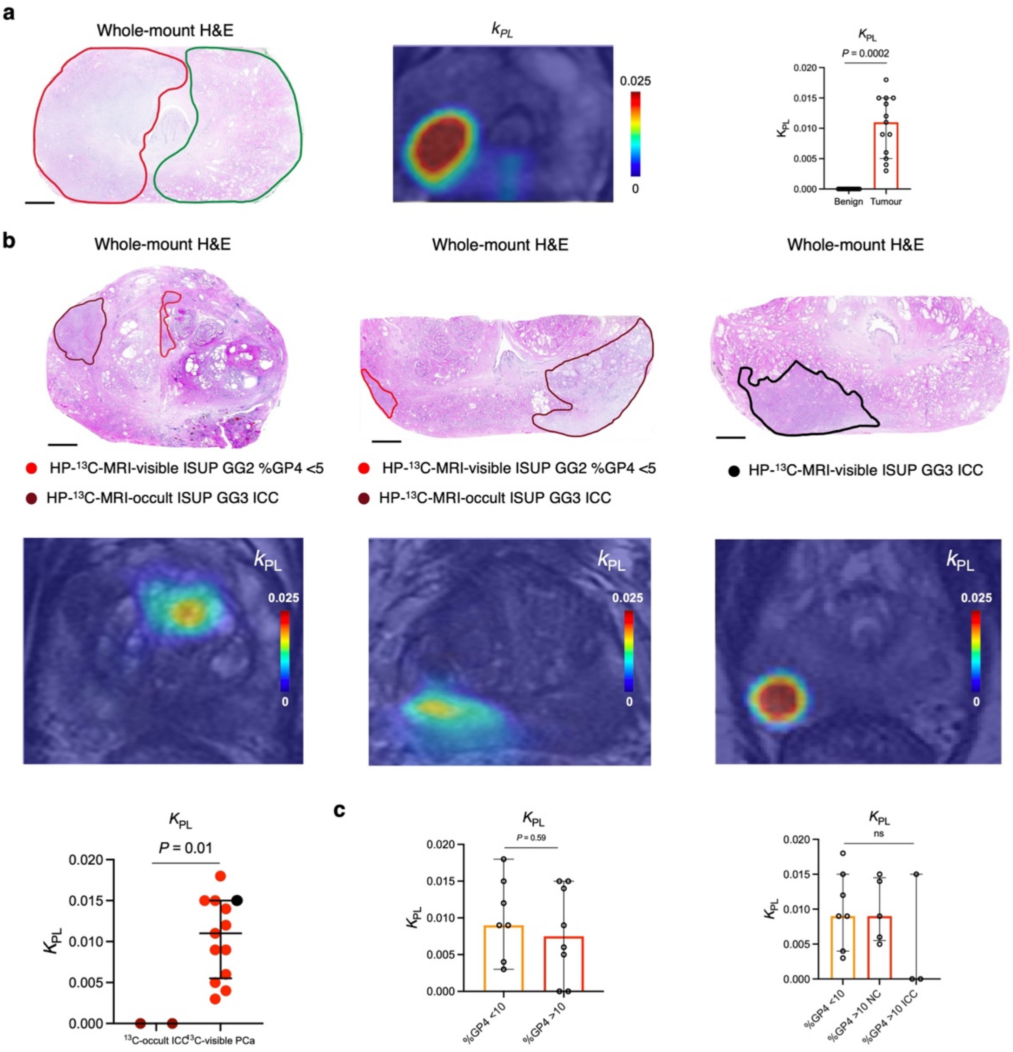

Supplementary Fig. 1. Assessment of the apparent reaction rate constant for the exchange of the hyperpolarised ^{13}C label between pyruvate and lactate (k_{PL}) in the benign and malignant prostate, as well as in patients with cribriform prostate cancer. a, Representative whole-mount H&E and k_{PL} map from the patient presented in **Fig. 2** of the main text showing increased k_{PL} in the tumour region (red outline on the H&E map) compared to the contralateral benign prostate (green outline on the H&E map), with the scatterplot with bars comparing k_{PL} values between the two tissue types. **b**, Whole-mount H&E and k_{PL} maps from patients presented in **Fig. 5** of the main text who harboured large cribriform tumours, two out of three of which showed an unmeasurable k_{PL}. A mixed box-and-whisker and scatterplot compares k_{PL} values between ^{13}C-occult and ^{13}C-visible lesions, with the k_{PL} value corresponding to the single case of a ^{13}C-visible cribriform tumour depicted in black. **c**, Scatterplots with bars comparing k_{PL} values between tumours divided based on the percentage (left) and histological subtype (right) of Gleason pattern 4 disease, similar to the analysis presented in **Fig. 6** of the main text. In **b**, the whole-mount H&E map on the left is a fusion of two separate slides, which individually represent two halves of the whole gland sectioned at the same level. *P* were derived using the Mann-Whitney U test. Scale bars in **a-b** denote 5mm.

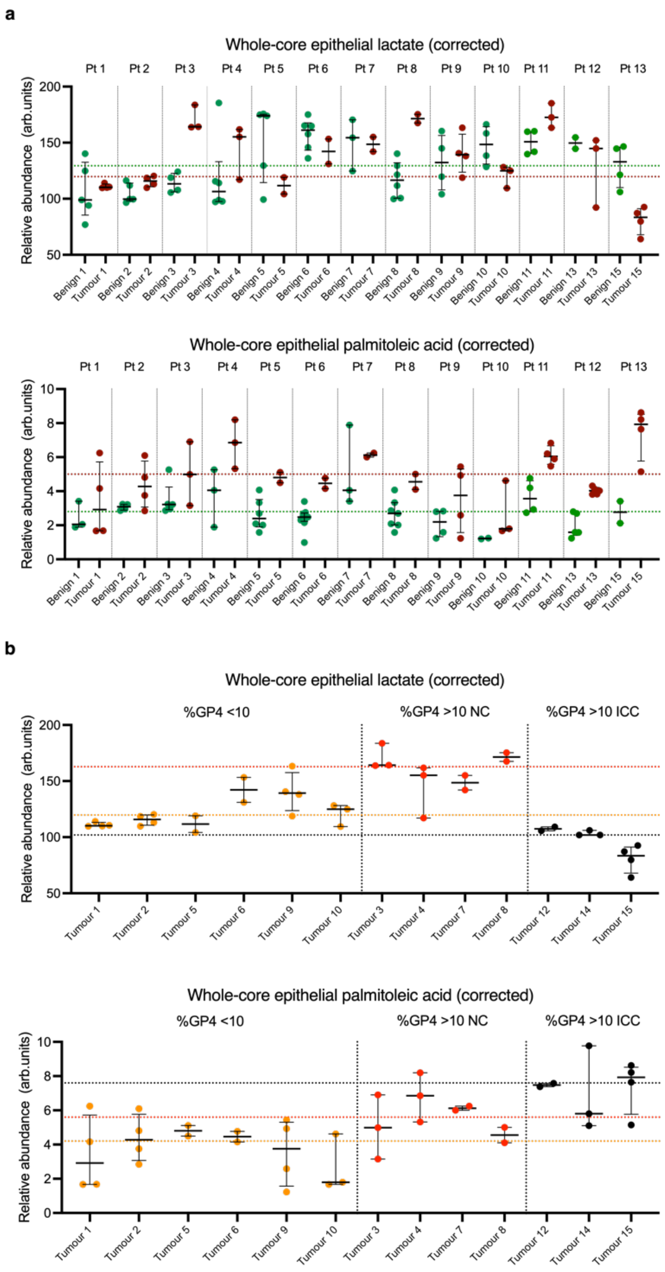

Supplementary Fig. 2. Visual representation of patient-to-patient variation in DESI-MSI-derived epithelial lactate and palmitoleic acid abundances in the benign and malignant prostate, as well as lesions of different histological phenotypes. a, Mixed box-and-whisker and scatterplots demonstrating patient-to-patient variation of whole-core epithelial lactate and palmitoleic acid abundances (both corrected for cell density), with green and red horizontal lines denoting median values corresponding to all benign and malignant samples, respectively. **b**, Mixed box-and-whisker and scatterplots presenting patient-to-patient variation of the same metabolite abundances, with tumours grouped as those with %GP4 <10 (orange), %GP4 >10 non-cribriform (NC; red), and %GP4 >10 invasive cribriform carcinoma (ICC; black), with corresponding median values presented as horizontal lines. Individual patient and lesion characteristics are summarised in **Table 1** of the main text, with the figure used to support the key findings presented in **Fig. 2** and **Fig. 7** of the main text.

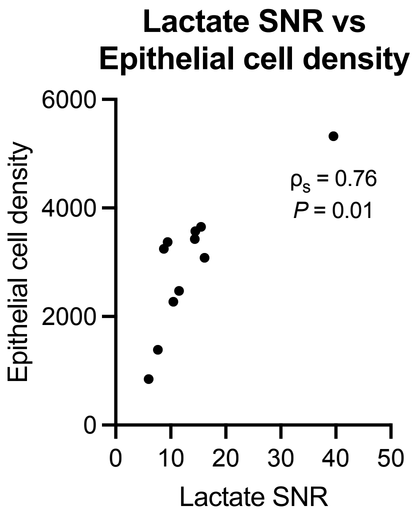

Supplementary Fig. 3. **Relationship between tumour epithelial cell density and clinical [1-^{13}C]lactate labelling.** Spearman's correlation plot comparing tumour-derived HP-^{13}C-MRI-derived [1-^{13}C]lactate SNR and digital-pathology-derived epithelial cell density quantified using the random forest tissue classifier described in the main manuscript and prior publications[1,2]. The plot includes the rank correlation coefficient and a corresponding *P* value. The analysis included data from n = 11 HP-^{13}C-MRI-visible lesions.

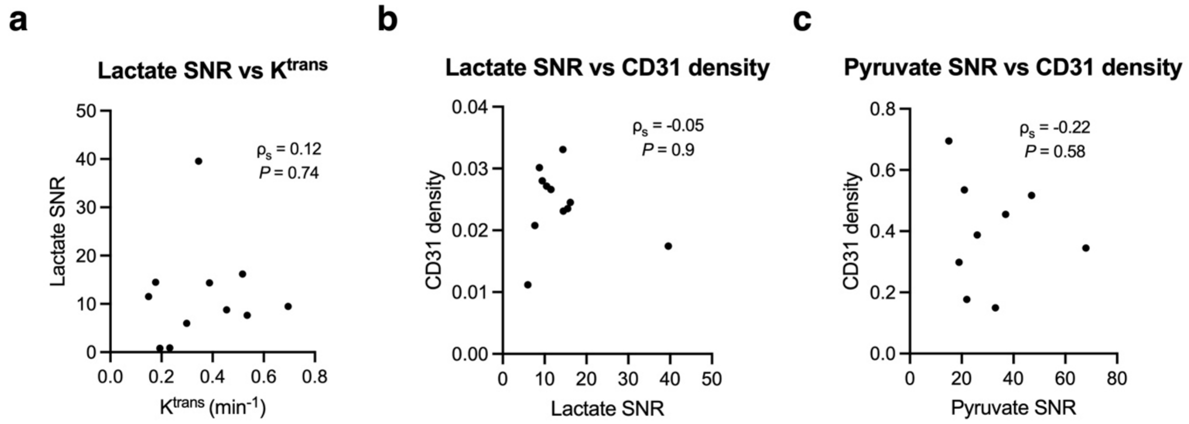

Supplementary Fig. 4. **Relationship between tumour vascular density, perfusion and permeability, and clinical [1-^{13}C]lactate or [1-^{13}C]pyruvate labelling.** **a,** Spearman's correlation plot comparing hyperpolarised tumour [1-^{13}C]lactate SNR and K^{trans} on ^{1}H-MRI. **b,** Spearman's correlation plot comparing hyperpolarised tumour [1-^{13}C]lactate SNR and tissue-based CD31 density. Both panels include individual rank correlation coefficients and corresponding *P* values. **c,** Spearman's correlation plot comparing hyperpolarised tumour [1-^{13}C]pyruvate SNR and tissue-based CD31 density**.** The lack of a significant relationship between tumour [1-^{13}C]pyruvate SNR and tumour K^{trans} has been previously reported in this cohort[1] (ρ_s = -0.14; P = 0.69). All analyses included data from n = 11 HP-^{13}C-MRI visible lesions.

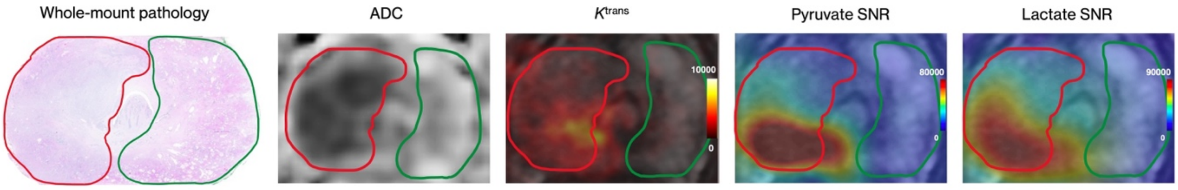

Supplementary Fig. 5. Segmentation of benign and malignant prostate areas in the HP-^{13}C-MRI cohort. Representative whole-mount pathology, ^{1}H-MRI-derived ADC and K^{trans} maps, along with HP-^{13}C-MRI-derived [1-^{13}C]pyruvate and [1-^{13}C]lactate SNR maps overlaid on conventional T_2-weighted images with overlaid tumour (red) and contralateral benign (green) ROIs. Drawn by an expert genitourinary pathologist, these ROIs were transposed onto IHC and RNAscope images to derive the expression of proteins and mRNA molecules-of-interest.

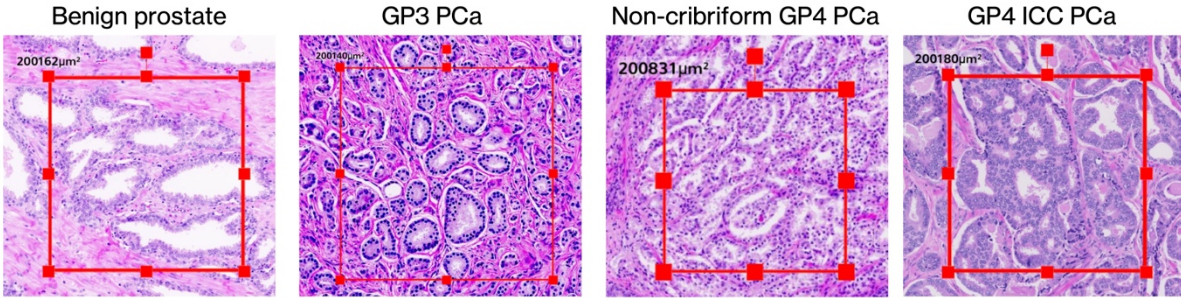

Supplementary Fig. 6. Single-gland segmentation of benign and malignant prostate in the HP-^{13}C-MRI cohort. Representative H&E images including standardised ROIs encompassing areas of pure benign prostatic epithelium, as well as GP3 PCa, non-cribriform GP4 disease (glomeruloid glands), and cribriform GP4 PCa (invasive cribriform carcinoma). These ROIs were also transposed onto IHC and RNAscope slides to derive biomarker expression specific to a particular histological entity.

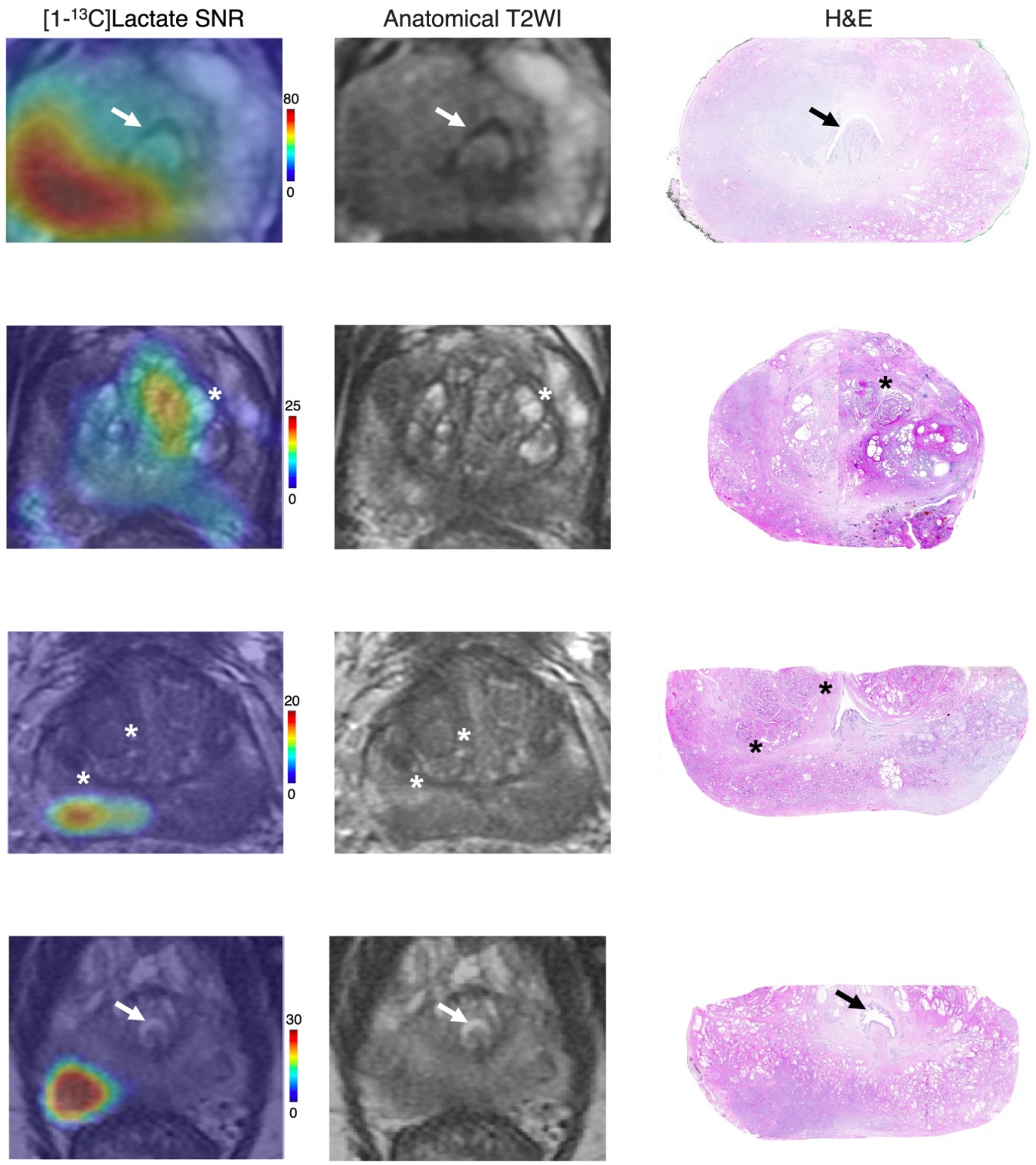

Supplementary Fig. 7. Anatomical matching of HP-^{13}C-MRI, ^{1}H-MRI, and whole-mount H&E images in the primary cohort. To ensure adequate matching of benign and malignant tissue ROIs included in this analysis involving direct comparison of several imaging modalities, we matched whole-mount H&E slices (right column) with anatomical T_2-weighted images (middle column) as described in the Methods sections of the main text. This figure illustrates matching of the location and shape of such anatomical landmarks as the urethra (white and black arrows) and pseudocapsules of benign hyperplastic nodules (white and black asterisks), as shown in four example cases. Anatomical matching of HP-^{13}C-MRI and ^{1}H-MRI, as illustrated in the image overlay in the left column, was performed simply by aligning the DICOM coordinates. The resulting matched benign and tumour ROIs are presented in **Supplementary Fig. 5**.

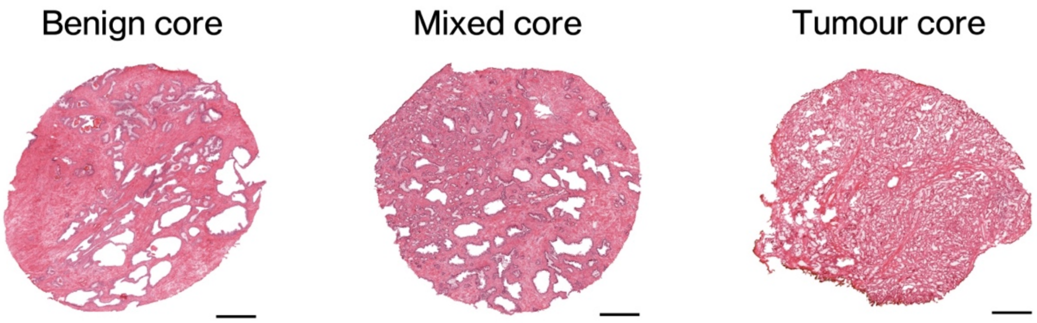

Supplementary Fig. 8. Fresh-frozen specimen classification in the DESI-MSI cohort. Representative H&E images of benign (0% tumour tissue), mixed (<50% tumour tissue), and tumour (>80% tumour tissue) fresh-frozen specimen from the spatial metabolomics cohort. Scale bars denote 1mm.

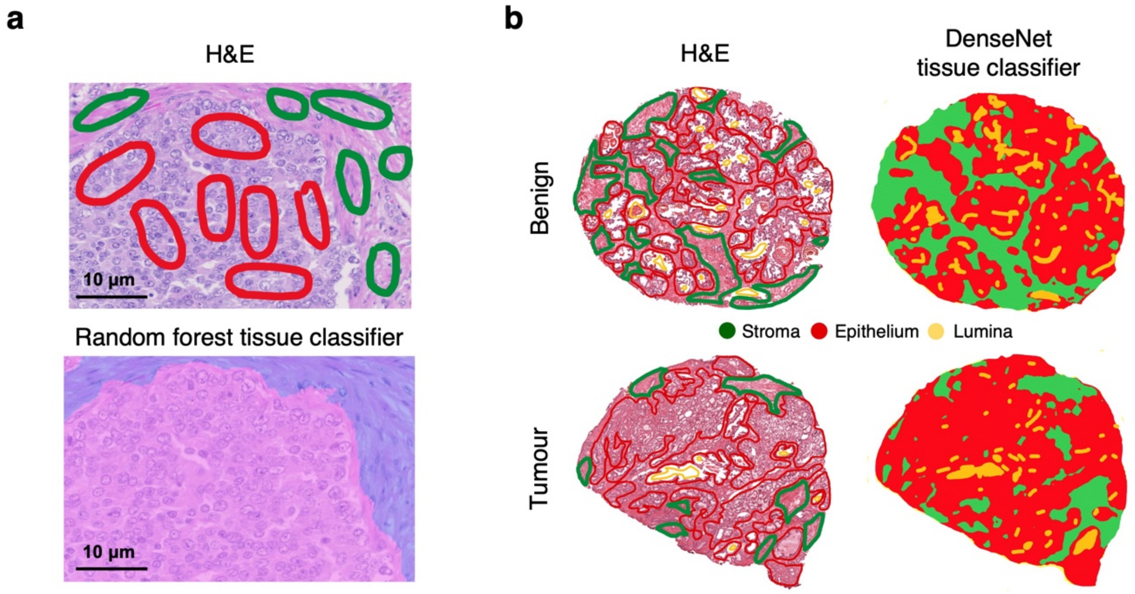

Supplementary Fig. 9. Tissue classifiers used in this study. a, H&E image with epithelial (red) and stromal (green) ROIs used to train the random forest classifier used in the HP-^{13}C-MRI cohort. **b,** H&E images with stromal (green), epithelial (red), and luminal (yellow) ROIs used to train the DenseNet tissue classifier used in the DESI-MSI cohort.

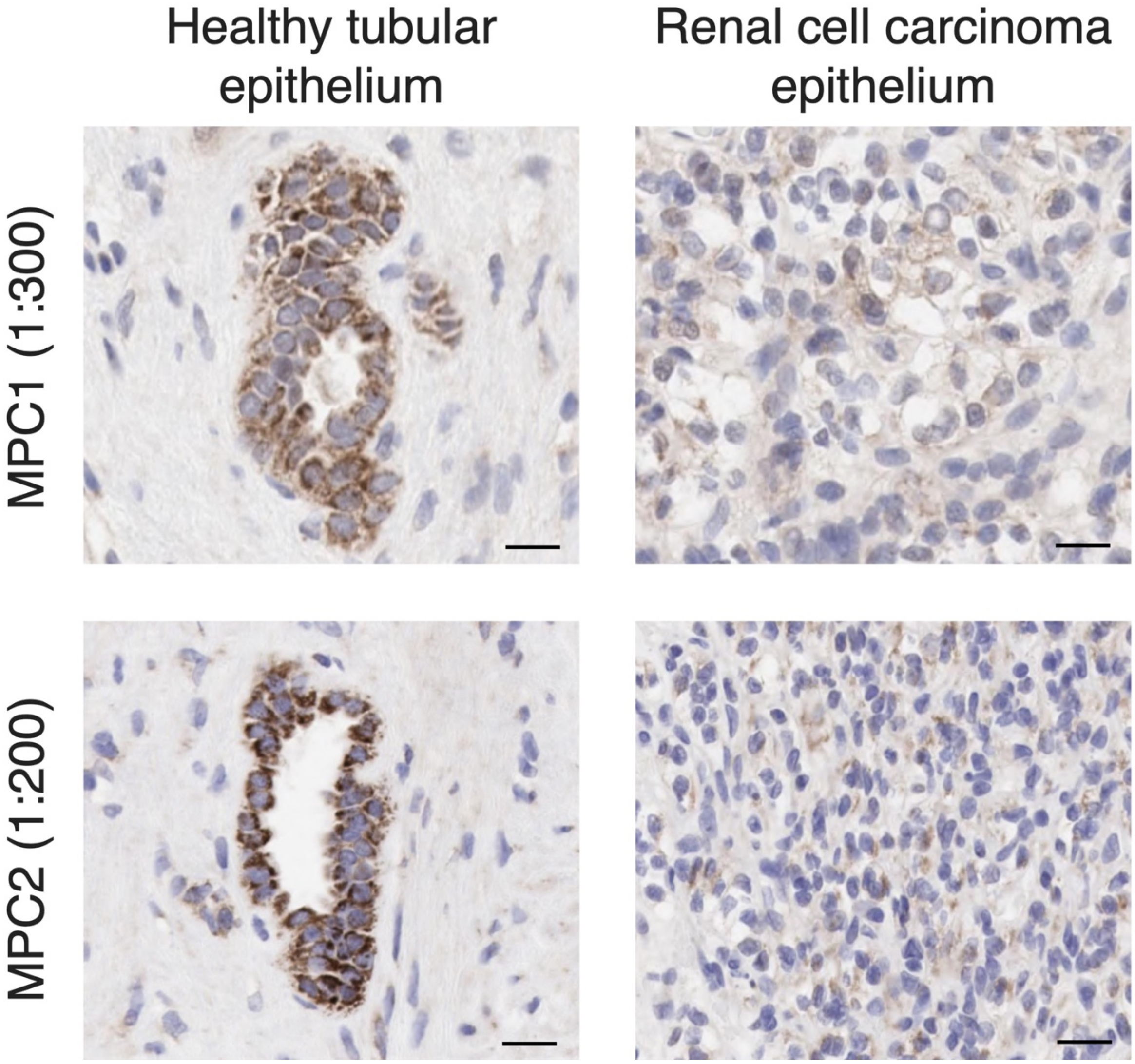

Supplementary Fig. 10. MPC1 and MPC2 antibody validation. For validation purposes, human kidney samples containing healthy tubular epithelium (left column) and foci of renal cell carcinoma (RCC; right column) were stained with MPC1 and MPC2 antibodies using several antibody titrations. As demonstrated by the relevant Human Protein Atlas entries, both MPC1 and MPC2 are overexpressed in healthy tubular epithelium compared to RCC epithelium, which we observed during the validation process as shown in these representative images. For MPC1, the 1:300 antibody titration was optimal, while for MPC2, the 1:200 antibody titration gave the best contrast between the two tissue types. Scale bars for images of healthy tubular epithelium denote 100 µm, and 50 µm for images of RCC epithelium.

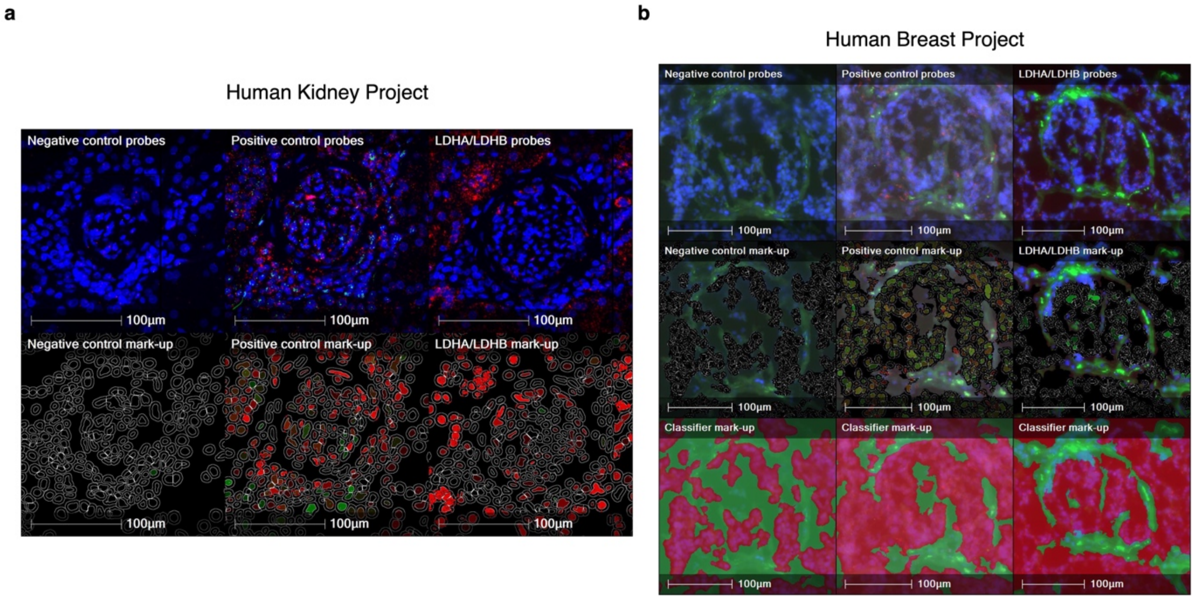

Supplementary Fig. 11. Example images of the *LDHA* and *LDHB* RNAscope probe validation conducted in human kidney and breast tissue samples. Scanned images of FFPE specimens from the two validation experiments are provided. In both experiments, the negative control slides had DapB *is* in every channel, while the positive control slides included *POLR2A* and *PPIB* probes. In panel **a**, fluorescent scanning was performed using Akoya PhenoImager HT, with subsequent images unmixed on InForm software to separate autofluorescence. In panel **b**, images were acquired using Zeiss widefield microscope, with the bottom sub-panel demonstrating the outputs of a classifier used to filter out autofluorescence.

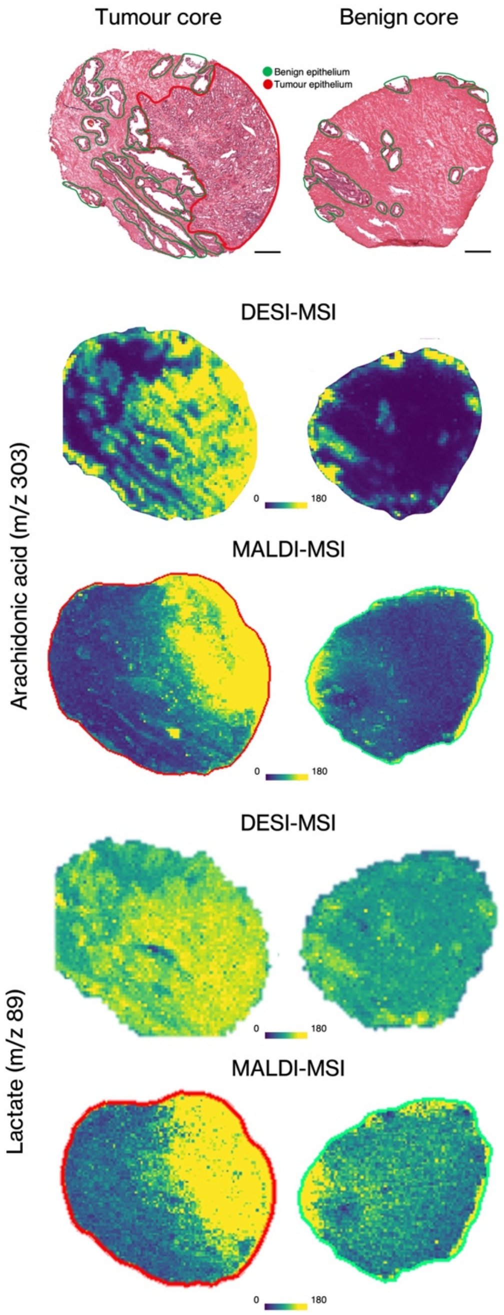

Supplementary Fig. 12. Direct comparison of DESI-MSI and MALDI-MSI outputs in benign and malignant prostatic cores from the secondary cohort. Representative H&E images of benign and tumour cores are provided, along with corresponding DESI-MSI and MALDI-MSI (negative ion mode) images demonstrating similar spatial distribution and comparable relative abundance of arachidonic acid (m/z 303) and lactate (m/z 89). The slight spatial differences between DESI-MSI and MALDI-MSI, best illustrated by the predominant localisation of both metabolites to tumour epithelial glands (red) compared to adjacent benign epithelium (green) can be explained by the ion suppression effect, which is indicative of different biophysical properties of the two tissue types and is more pronounced in MALDI-MSI compared to DESI-MSI[3,4]. Direct comparison between the two techniques is further complicated by the matrix effects specific to MALDI-MSI, different normalisation methods used for the two mass analysers, and imperfect adjustment of signal intensities in the Scils software.

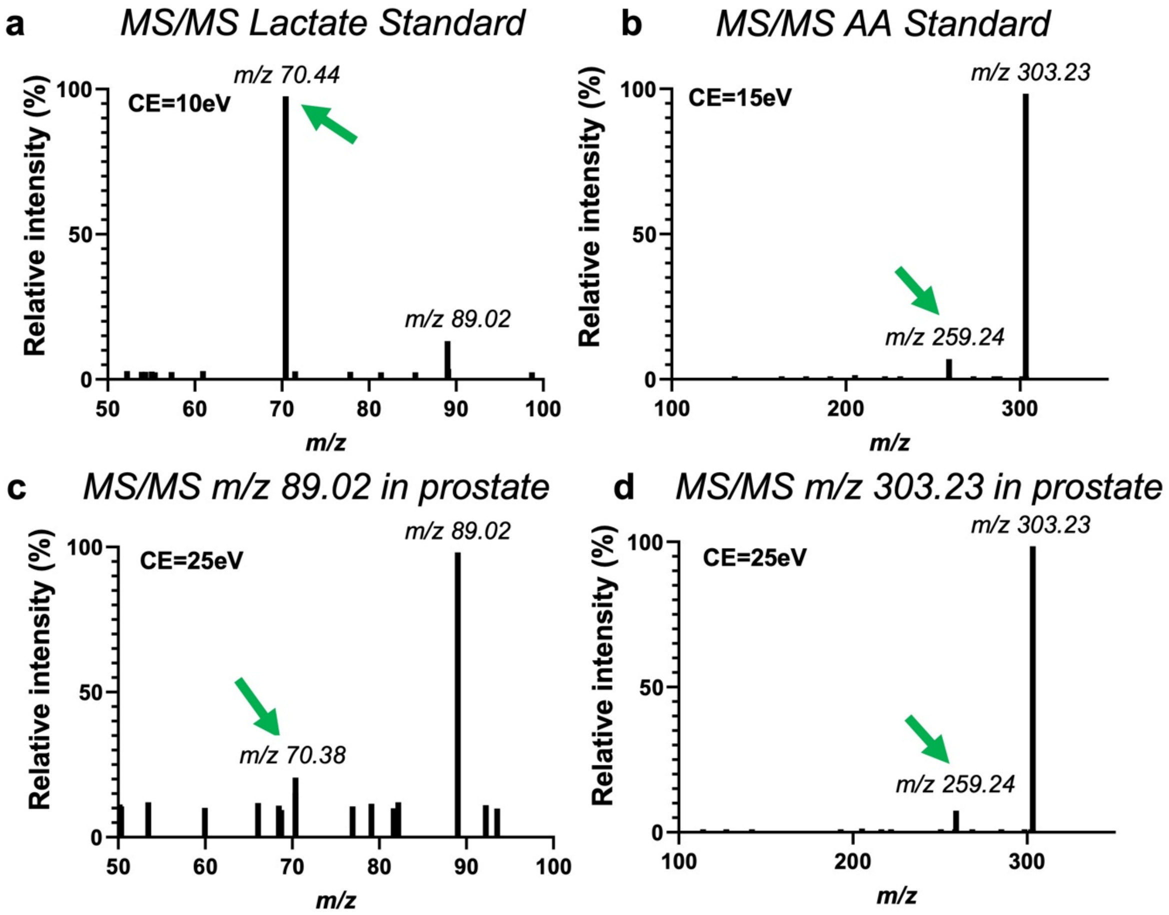

Supplementary Fig. 13. Confirmation of lactate and arachidonic acid (AA) detection in human prostate tissues using analytical standards. Standards for lactate (a), and AA (b) were applied to a target slide and a DESI MS/MS spectrum (negative ion mode) was collected using collision-induced dissociation (CID) with a nominal collision energy (eV) adjusted for each metabolite and condition. Parent m/z values observed in the standards were subsequently evaluated in the human prostate tissue sections (c, d). Metabolite fragments observed in the tissue were compared to those obtained from the standards. Green arrows in each spectrum indicate characteristic fragment peaks that were similar between the standards and human prostate samples. Identification of fragments from small metabolites such as lactate can be challenging due to the lower intensity of the parent peak and the limited fragmentation pattern on a high background MS/MS spectrum as demonstrated in the prostate specimen in panel (c). This contrasts with the large peak from a large and abundant metabolite such as AA, which demonstrates a higher signal-to-noise ratio on a low background MS/MS spectrum as seen in panel (d).

Supplementary References:

1. Sushentsev, N. *et al.* Hyperpolarised 13C-MRI identifies the emergence of a glycolytic cell population within intermediate-risk human prostate cancer. *Nat. Commun. 131* **13**, 1–12 (2022).

2. Brodie, C. Overcoming Autofluorescence (AF) and Tissue Variation in Image Analysis of In Situ Hybridization. *Methods Mol. Biol.* **2148**, 19–32 (2020).

3. Hamm, G. *et al.* Quantitative mass spectrometry imaging of propranolol and olanzapine using tissue extinction calculation as normalization factor. *J Proteomics* 30; **75(16**):4952-4961 (2012).

4. Taylor, AJ. *et al*. Exploring Ion Suppression in Mass Spectrometry Imaging of a Heterogeneous Tissue. *Anal Chem.* 90 (**9**):5637-5645 (2018).
