# Supplementary material for: Metabolic imaging across scales reveals distinct prostate cancer phenotypes
Source: Nat Commun. 2024 Jul 16;15:5980. doi: 10.1038/s41467-024-50362-5 (PMC11252279; doi:10.1038/s41467-024-50362-5)
Supplement: Supplementary file 3 — Reporting Summary [file 41467_2024_50362_MOESM3_ESM.pdf]

nature portfolio

Corresponding author(s): Nikita Sushentsev
Last updated by author(s): May 27, 2024

Reporting Summary

Nature Portfolio wishes to improve the reproducibility of the work that we publish. This form provides structure for consistency and transparency in reporting. For further information on Nature Portfolio policies, see our Editorial Policies and the Editorial Policy Checklist.

Statistics

For all statistical analyses, confirm that the following items are present in the figure legend, table legend, main text, or Methods section.

n/a	Confirmed	
☐	☒	The exact sample size (*n*) for each experimental group/condition, given as a discrete number and unit of measurement
☐	☒	A statement on whether measurements were taken from distinct samples or whether the same sample was measured repeatedly
☐	☒	The statistical test(s) used AND whether they are one- or two-sided<br>*Only common tests should be described solely by name; describe more complex techniques in the Methods section.*
☒	☐	A description of all covariates tested
☐	☒	A description of any assumptions or corrections, such as tests of normality and adjustment for multiple comparisons
☐	☒	A full description of the statistical parameters including central tendency (e.g. means) or other basic estimates (e.g. regression coefficient) AND variation (e.g. standard deviation) or associated estimates of uncertainty (e.g. confidence intervals)
☐	☒	For null hypothesis testing, the test statistic (e.g. *F*, *t*, *r*) with confidence intervals, effect sizes, degrees of freedom and *P* value noted<br>*Give P values as exact values whenever suitable.*
☒	☐	For Bayesian analysis, information on the choice of priors and Markov chain Monte Carlo settings
☒	☐	For hierarchical and complex designs, identification of the appropriate level for tests and full reporting of outcomes
☒	☐	Estimates of effect sizes (e.g. Cohen's *d*, Pearson's *r*), indicating how they were calculated

Our web collection on statistics for biologists contains articles on many of the points above.

Software and code

Policy information about availability of computer code

Data collection	Proton and hyperpolarised MRI were acquired using commercially available software based on the GE Healthcare platform used for these studies.
Data analysis	Hyperpolarised MRI data were reconstructed in MATLAB R2018b (The MathWorks, Inc., Natick, MA).<br>Benign and tumour regions of interest (ROIs) were generated using OsiriX 10.0 (Pixmeo SARL, Bernex, Switzerland).<br>HALO v3.2.1851.266 (Indica Labs, Albuquerque, NM, USA) was used for immunohistochemistry and RNAscope analyses.<br>Statistical analyses were performed in GraphPad Prism (version 9.0.2, GraphPad Software, San Diego, CA, USA).<br>The code to build a metabolic tissue classifier is available at (https://github.com/AstraZeneca/metabolic_classifier v1.0). The code to conduct metabolic pathway enrichment analysis is available at (https://github.com/AleksZakirov/MPE-analysis-for-prostate-cancer-study v1.0).

For manuscripts utilizing custom algorithms or software that are central to the research but not yet described in published literature, software must be made available to editors and reviewers. We strongly encourage code deposition in a community repository (e.g. GitHub). See the Nature Portfolio guidelines for submitting code & software for further information.

Data

Policy information about availability of data

All manuscripts must include a data availability statement. This statement should provide the following information, where applicable:

- Accession codes, unique identifiers, or web links for publicly available datasets
- A description of any restrictions on data availability
- For clinical datasets or third party data, please ensure that the statement adheres to our policy

The authors declare that the clinical and imaging data supporting the findings of this study are available within the article and its Supplementary Information. The open-source TCGA-PRAD data used in this study are available through the NCI GDC Data Portal, with additional single-cell and spatial RNA-sequencing analyses performed on two publicly available datasets, EGAS00001005787 and GSE176031, respectively. Data used to generate plots in Fig. 2-7 along with the DESI-MSI metabolite data are provided in the Source Data file. The authors defer raw DESI-MSI and clinical MRI data deposition to ensure compliance with legal requirements of the University of Cambridge and Cambridge University Hospitals NHS Foundation Trust and avoid compromising privacy of the study participants. Requests for raw data can be referred to the corresponding author (N.S.); these will be reviewed within ten working days in consultation with the institutional R&D which will determine the terms of a data transfer agreement between the recipient institution, the University of Cambridge, and Cambridge University Hospitals NHS Foundation Trust.

Research involving human participants, their data, or biological material

Policy information about studies with human participants or human data. See also policy information about sex, gender (identity/presentation), and sexual orientation and race, ethnicity and racism.

Reporting on sex and gender	Due to the prostate cancer-specific focus of this study, it only included male patients whose sex was specified in their clinical records.
Reporting on race, ethnicity, or other socially relevant groupings	We have not reported any of these categories in our manuscript.
Population characteristics	This study included two prospective cohorts of prostate cancer patients who underwent robot-assisted radical prostatectomy in our centre. The study included a total of 21 patients (median age 67 years, interquartile range 63–69 years) who harboured a total of 30 biopsy-proven prostate tumours. The study population included two matched prospective surgical cohorts who were matched based on tumour histopathological characteristics, as outlined in Table 1 of the main manuscript.
Recruitment	Patients in both cohorts were selected at clinical multi-disciplinary team meetings or clinics and approached by clinical staff involved in their routine care. If patients agreed, they were then approached by the research staff when the nature of the two studies (MISSION-Prostate or DIAMOND, as detailed in the below section) was explained and they were given written information to read. Patients were given at least 24 hours to consider the study before consenting.
Ethics oversight	All patients from the hyperpolarised MRI cohort provided written consent to participate in the original prospective study (Molecular Imaging and Spectroscopy with Stable Isotopes in Oncology and Neurology – Imaging metabolism in prostate [MISSION-Prostate] protocol), retrospective analysis of which was approved by the institutional review board (National Research Ethics Service Committee East of England, Cambridge South, Research Ethics Committee number 16/EE/0205).<br>All patients from the spatial metabolomics cohort provided written informed consent to participate in an ethically-approved national study (DIAMOND, National Research Ethics Service Committee East of England, Cambridge South, Research Ethics Committee number 03/018), the protocol of which enabled biological data analyses reported in this manuscript.

Note that full information on the approval of the study protocol must also be provided in the manuscript.

Field-specific reporting

Please select the one below that is the best fit for your research. If you are not sure, read the appropriate sections before making your selection.

[x] Life sciences   [ ] Behavioural & social sciences   [ ] Ecological, evolutionary & environmental sciences

For a reference copy of the document with all sections, see nature.com/documents/nr-reporting-summary-flat.pdf

Life sciences study design

All studies must disclose on these points even when the disclosure is negative.

Sample size	For the purpose of this pilot physiological study, with the primary objective of identifying biological mechanisms underpinning differential [1-13C]lactate labelling between the benign and malignant prostate, as well as between cribriform and non-cribriform intermediate-risk tumours, insufficient prior knowledge is available to perform a formal sample size calculation. The defined sample size of the MISSION-Prostate study has therefore been determined based on pragmatic considerations of the anticipated recruitment rates over the specified study duration, with the final number of included patients impacted severely by the COVID-19 pandemic that led to the cancellation of all routine imaging studies. The size of the matched DIAMOND cohort was determined by the number and histopathological characteristics of primary tumours (n = 15) available for analysis as part of the MISSION-Prostate study.
Data exclusions	In the hyperpolarised MRI cohort, two tumours were excluded from the imaging analysis due to technical failure of the HP 13C-MRI. Tissue

	samples from one patient were excluded from the analysis based on the impact of the interval androgen deprivation therapy on physiological mechanisms investigated in this study. This exclusion could not be foreseen because the interval androgen deprivation therapy was prescribed due to cancellation of all elective surgeries due to COVID-19 pandemic and was, therefore, not part of a standard of care. These data exclusions are described in the manuscript.
Replication	The hyperpolarised imaging data was acquired following a single injection and could not be repeated. However, images were acquired at multiple timepoints following injection which reduced the effects of noise or artefact in a single image. IHC, DESI-MSI, and RNAscope experiments were not repeated, however, extensive validation of the methods was conducted as described in the main manuscript and elsewhere in this Reporting Summary.
Randomization	Randomization was not undertaken as there was no therapeutic intervention. Consecutive patients fulfilling the required inclusion and exclusion criteria and consenting to be enrolled were included to avoid investigator bias. Patient selection in the DIAMOND cohort was guided by specific histopathological characteristics of tumours derived from the primary cohort, as described in the main text.
Blinding	The IHC, RNAscope, and DESI-MSI data were analyzed in a blinded fashion. The clinical clinical data was anonymized prior to analysis although given the small patient size, this was not blinded.

Reporting for specific materials, systems and methods

We require information from authors about some types of materials, experimental systems and methods used in many studies. Here, indicate whether each material, system or method listed is relevant to your study. If you are not sure if a list item applies to your research, read the appropriate section before selecting a response.

Materials & experimental systems

n/a	Involved in the study
☐	☒ Antibodies
☒	☐ Eukaryotic cell lines
☒	☐ Palaeontology and archaeology
☒	☐ Animals and other organisms
☐	☒ Clinical data
☒	☐ Dual use research of concern
☒	☐ Plants

Methods

n/a	Involved in the study
☒	☐ ChIP-seq
☒	☐ Flow cytometry
☒	☐ MRI-based neuroimaging

Antibodies

Antibodies used	MCT1 (Cat. No. HPA003324, Atlas Antibodies, Bromma, Sweden)<br>MPC1 and MPC2 (Cat. No. PAB28306, Abnova, Taipei, Taiwan for MPC1; Cat. No. D417G, Cell Signaling Technology, Danvers MA, USA for MPC2)<br>FASN (Cat. No. 3180, Cell Signaling Technology, Danvers MA, USA)<br>AR (Cat. No. NCL-AR-318, Novocastra, Newcastle, UK)<br>HIF-1α (Cat. No. ab51608, Abcam, Cambridge, UK)<br>CD31 (Cat. No. M0823, Dako, Santa Clara CA, USA)<br>Hs-LDHA-C1 (Cat. No. 487818, ACD Bio-Techne, Abingdon, UK)<br>Hs-LDHB-C2 (Cat. No. 531278-C2, ACD Bio-Techne, Abingdon, UK)
Validation	All antibodies have been previously validated in our centre using positive and negative tissue controls under the supervision of specialist pathologists.<br><br>For the MCT1 antibody validation, we initially tested the antibody in a brain cell line that served as a positive control. We tested the antibody at a single dilution (1:100) with three different pre-treatments (sodium citrate, Tris EDTA, and enzyme), along with a no primary control for each retrieval. We saw strong cell surface signal with both sodium citrate and Tris EDTA, as expected MCT1 which is most commonly localised at cell membrane (predominant cell membrane staining in the prostate samples can be clearly seen in Fig. 2i and Fig. 5d of the main text). The initial tissue tests of the antibody were subsequently carried out in a human xenograft in a rat model (Rat 3(2)_SP13) with a range of antibody titrations. Following the review of positive and negative control sections by a consultant neuropathologist, the antibody dilution to 23.36 µg/mL was deemed optimal; this was subsequently confirmed in primary human tissue. The antibody has been used routinely since it was validated in 2014.<br>For CD31, we tested the antibody at a single dilution (1:50) with three different pre-treatments (sodium citrate, Tris EDTA, and enzyme) in FFPE human tonsil, along with a no primary control for each retrieval condition. Sodium citrate pre-treatment was taken forward and tested on FFPE human breast cancer TMA offcuts. Results were reviewed by an expert human breast pathologist, with optimal conditions (of a 1:50 antibody dilution and sodium citrate antigen retrieval buffer) agreed. The antibody has been used routinely since it was validated in 2018. Specific vascular staining is clearly demonstrated in Fig. 2i and Fig. 5d on the main text.<br>For FASN, we tested the antibody at a single dilution (1:100) with three different pre-treatments (sodium citrate, Tris EDTA, and enzyme) in C42b (a FAS over-expressing) and a FAS knockdown FFPE human cell-line, with a no primary control for each retrieval. Sodium citrate and Tris EDTA antigen retrieval yielded positive staining so both antigen retrieval conditions were tested in FFPE human prostate TMA off-cuts next, again including a no primary control for each retrieval condition. Tris EDTA was settled upon as giving the best result with positive staining in prostate cancer tissue and negative staining in normal prostate. The antibody has been used routinely since it was validated in 2011. Cytosolic staining is clearly demonstrated in Fig. 3b, Fig. 5d, and Fig. 6c of the main text.

For AR, we tested the antibody at two dilutions (1:100 & 1:250) with two different pre-treatments (sodium citrate & Tris EDTA) in FFPE LNCaps (a human prostate cell line), along with a no primary control for each retrieval condition. Tris EDTA pre-treatment only was taken forward with a 1:50, 1:100 & 1:250 dilution on FFPE human prostate cancer TMA offcuts, alongside the LNCaps, and with no primary controls. Optimal conditions of a 1:50 antibody dilution and Tris EDTA antigen retrieval were settled upon in agreement with an expert genitourinary pathologist. The antibody has been used routinely since it was validated in 2008. Exclusively nuclear staining is clearly demonstrated in Fig. 3b, Fig. 5d, and Fig. 6c of the main text. Importantly, in Fig. 5b, nuclear AR staining is exclusively seen in luminal epithelial cells in the benign gland, with no staining detected in basal epithelium.

For HIF-1a, we tested the antibody at a single dilution (1:100) with three different pre-treatments (sodium citrate, Tris EDTA, and enzyme) in FFPE human breast tissue (normal versus tumour), with a no primary control for each retrieval. Sodium citrate and Tris EDTA antigen retrieval yielded positive staining so both antigen retrieval conditions were tested in FFPE human breast TMA off-cuts next using a dilution of 1:50 & 1:100 for sodium citrate and 1:100 & 1:200 for Tris EDTA. Results were reviewed by an expert human breast pathologist and the antibody dilution of 1:100 (23.36 μg/mL) with sodium citrate retrieval deemed optimal as it differentiated positive and negative cases clearly. The antibody has been used routinely since it was validated in 2018. Both cytosolic and nuclear staining is clearly demonstrated in Fig. 6c of the main text.

For the RNAscope experiment, prior to the analysis, we used spare tissue sections to run the negative control slides (4 Plex DapB to ensure that DapB is in every channel) to assess background staining, along with the positive control slides (POLR2A for channel 1 and PPIB for channel 2) to determine good RNA quality. In the analysis optimisation, we used the negative controls to set the thresholds for positive signal in the test slides. The described routine in-house RNAscope antibody validation process, along with the subsequent analysis, was performed by an experienced member of our dedicated Histopathology Core Facility (see Acknowledgments) with 11-years' experience of using all RNAscope automated kits available for the Leica Bond Rx (Single Plex, Duplex, 3 Plex, 4 Plex, BaseScope, and RNAscope Plus), as well as manual HiPlex kits, for more than 50 separate projects in a variety of tissues and species, including human and murine breast, brain, kidney, lung, and liver.

Clinical data

Policy information about clinical studies

All manuscripts should comply with the ICMJE guidelines for publication of clinical research and a completed CONSORT checklist must be included with all submissions.

Clinical trial registration	This study falls under the remit of a physiological study rather than a formal clinical trial. This has been approved by the Medicines and Healthcare products Regulatory Agency (MHRA) in the UK.
Study protocol	Both MISSION-Prostate and DIAMOND study protocols will be made available on a local publicly available site at the close of the study.
Data collection	Both MISSION-Prostate and DIAMOND are single-centre recruiting studies. The data are presented from patients recruited between May 2018 and February 2020 (MISSION-Prostate) and May 2016 and November 2016 (DIAMOND).
Outcomes	Since this is a physiological feasibility study, all outcomes were exploratory, including understanding of specific biological mechanisms behind the differential [1-13]lactate labelling between the benign and malignant prostate, as well as cribriform and non-cribriform intermediate-risk tumours.

Plants

Seed stocks	N/A
Novel plant genotypes	N/A
Authentication	N/A
