# Supplementary material for: Metabolic imaging across scales reveals distinct prostate cancer phenotypes
Source: Nat Commun. 2024 Jul 16;15:5980. doi: 10.1038/s41467-024-50362-5 (PMC11252279; doi:10.1038/s41467-024-50362-5)
Supplement: Supplementary file 4 — Inventory of Supporting Information [file 41467_2024_50362_MOESM4_ESM.docx]

**Metabolic imaging across scales reveals distinct prostate cancer phenotypes**

Nikita Sushentsev^1†^*, Gregory Hamm^2†^, Lucy Flint^2^, Daniel Birtles^2^, Aleksandr Zakirov^3^, Jack Richings^2^, Stephanie Ling^2^, Jennifer Y. Tan^2^, Mary A. McLean^1,4^, Vinay Ayyappan^1^, Ines Horvat Menih^1^, Cara Brodie^4^, Jodi L. Miller^4^, Ian G. Mills^5,6,7,8^, Vincent J. Gnanapragasam^9, 10,11^, Anne Y. Warren^12^, Simon T. Barry^13^, Richard J.A. Goodwin^2††^, Tristan Barrett^1††^, Ferdia A. Gallagher^1††^

^1^ Department of Radiology, Addenbrooke’s Hospital and University of Cambridge, Cambridge, UK

^2^ Imaging and Data Analytics, Clinical Pharmacology & Safety Sciences, R&D, AstraZeneca, Cambridge, UK

^3^ Department of Clinical Neurosciences, University of Cambridge, Cambridge, UK

^4^ Cancer Research UK Cambridge Institute, University of Cambridge, Cambridge, UK

^5^ Patrick G Johnston Centre for Cancer Research, Queen’s University Belfast, Belfast, UK

^6^ Nuffield Department of Surgical Sciences, University of Oxford, John Radcliffe Hospital, Oxford, UK

^7^ Centre for Cancer Biomarkers, University of Bergen, Bergen, Norway

^8^ Department of Clinical Science, University of Bergen, Bergen, Norway

^9^ Department of Urology, Cambridge University Hospitals NHS Foundation Trust, Cambridge, UK

^10^ Division of Urology, Department of Surgery, University of Cambridge, Cambridge, UK

^11^ Cambridge Urology Translational Research and Clinical Trials Office, Cambridge Biomedical Campus, Addenbrooke’s Hospital, Cambridge, UK

^12^ Department of Pathology, Cambridge University Hospitals NHS Foundation Trust, Cambridge, UK

^13^ Bioscience, Discovery, Oncology R&D, AstraZeneca, Cambridge, UK

^†^ These authors contributed equally

^††^ These authors jointly supervised the work

*Corresponding author. Email: ns784@medschl.cam.ac.uk

**Inventory of Supporting Information**

**Supplementary Information** file includes the following items:

Supplementary Tables 1-2

Supplementary Figures 1-13
